# Human values and beliefs and concern about climate change: a Bayesian longitudinal analysis

**DOI:** 10.1007/s11135-017-0538-z

**Published:** 2017-07-25

**Authors:** Gabriele Prati, Luca Pietrantoni, Cinzia Albanesi

**Affiliations:** Dipartimento di Psicologia, Università di Bologna, Viale Europa 115, 47521 Cesena, FC Italy

**Keywords:** Values, Climate change, Beliefs, Concern, Risk perception, Longitudinal, Bayesian

## Abstract

The aim of this study was to investigate the influence of human values on beliefs and concern about climate change using a longitudinal design and Bayesian analysis. A sample of 298 undergraduate/master students filled out the same questionnaire on two occasions at an interval of 2 months. The questionnaire included measures of beliefs and concern about climate change (i.e., perceived consequences, risk perception, and skepticism) and human values (i.e., the Portrait Values Questionnaire). After controlling for gender and the respective baseline score, universalism at Time 1 was associated with higher levels of perceived consequences of climate change and lower levels of climate change skepticism. Self-direction at Time 1 predicted Time 2 climate change risk perception and perceived consequences of climate change. Hedonism at Time 1 was associated with Time 2 climate change risk perception. The other human values at Time 1 were not associated with any of the measures of beliefs and concern about climate change at Time 2. The results of this study suggest that a focus on universalism and self-direction values seems to be a more successful approach to stimulate public engagement with climate change than a focus on other human values.

## Introduction

Climate change represents a threat to human society and ecosystems (Intergovernmental Panel on Climate Change 2014). Climate change mitigation requires global collective action and public engagement (Bernauer 2013). People show varying levels of engagement with climate change (Leiserowitz et al. 2013). There are differences among people, such that some people tend to be concerned about the existence of climate change whereas others tend to be skeptical about the reality and severity of climate change (Whitmarsh 2011). In addition, previous research has identified differences with respect to the extent to which people feel threatened (risk perception) and perceive the consequences associated with climate change (Bostrom et al. 2012; Leiserowitz 2006). In summary, the beliefs and concern about climate change are reflected in varying levels of perceived consequences of climate change, climate change risk perception and climate change skepticism (Whitmarsh 2011; Bostrom et al. 2012; Leiserowitz 2006; Leiserowitz et al. 2013).

Human values play an important role in in shaping beliefs and concern about climate change (O’Brien and Wolf 2010; O’Brien 2009). According to Corner et al. (2014), “disagreements about climate change are more likely to be about values than about the underlying science.” In brief, their theory is that human values play an important role in driving much of the political and cultural debate that has developed around climate change.

### Human values and beliefs and concern about climate change

According to Schwartz (1992), human values are defined as “a desirable transsituational goal varying in importance, which serves as a guiding principle in the life of a person or other social entity.” Human values are conceptualized as relatively stable dimensions although they may change in response to socio-cultural changes (Inglehart 2008) or extreme events (Prati and Zani 2013). Although different conceptualizations of values co-exist in the literature, the Value Theory (Schwartz 1992, 1994) has been dominant in social psychology and is considered useful in examining values in relation to climate change (O’Brien and Wolf 2010). Specifically, the Value Theory (Schwartz 1992, 1994) posits that there are four universal distinct clusters that structure ten motivational value types. These four higher order value types are organized in two bipolar dimensions: openness to change (hedonism, self-direction, and stimulation) versus conservation (security, tradition, and conformity), and self-enhancement (hedonism, achievement, and power) versus self-transcendence (universalism and benevolence).

According to the spillover hypothesis (Thøgersen and Crompton 2009; Scott 1977), positive engagement with climate change may transfer from one aspect to another in people’s life (Corner et al. 2014). The spillover hypothesis is derived from self-perception theory (Bem 1972) which postulates that the engagement in pro-environmental behavior makes pro-environmental values and norms more salient. Since pro-environmental behaviors can be performed for environmental concern as well as for economic gain, Corner et al. (2014) postulates that a focus on self-enhancing values will make behavioral ‘spillover’ less likely because personal gain (e.g., economic) was the main incentive for the behavior. On the contrary, the salience of self-transcendent values in the general population will promote engagement with climate change on different aspects of the issue due to the spillover effect. Based on this theory, Corner et al. (2014) concluded that self-transcendence values are expected to be strongly associated with positive engagement with climate change, while self-enhancing values appear less congruent with positive engagement with climate change. Thus, it seems reasonable to assume that self-transcendence values would be positively related to higher levels of environmental concern, specifically about climate change, while self-enhancing values would be related to lower levels of concern about climate change.

#### **Hypothesis 1**

Universalism and benevolence will be associated with higher levels of perceived consequences of climate change and climate change risk perception and lower levels of climate change skepticism.

#### **Hypothesis 2**

Hedonism, achievement, and power will be associated with lower levels of perceived consequences of climate change and climate change risk perception and higher levels of climate change skepticism.

While there are theoretical reasons to expect the self-transcendent versus self-enhancement dimension to be related to beliefs and concern about climate change, the role of openness to change (values that stress independence, such as self-direction and stimulation) versus conservatism (values that emphasize tradition and conformity) dimension is less clear from a theoretical standpoint and produced inconsistent results (e.g., de Groot and Steg 2008, 2010; Corner et al. 2014; Stern and Dietz 1994; Nordlund and Garvill 2002). Therefore, we formulate an open research question:

Will security, conformity, tradition, stimulation, and self-direction values be associated with perceived consequences of climate change, climate change skepticism, and climate change risk perception?

Most of the literature on values and beliefs and concern about climate change is based on cross-sectional studies, which limit attempts to determine cause-effect relationships. The usual line of argument of the literature conceptualizes human values as antecedents of beliefs and concern about climate change. Although there are strong theoretical arguments that values could be considered antecedents, by the use of a longitudinal design it is possible to draw firmer conclusions about the direction of causality than has been possible in previous cross-sectional research. In addition, common method variance is more likely to bias the results of specific relationships in cross-sectional studies compared to longitudinal studies (Podsakoff et al. 2012). Specifically, measurement context effects (i.e., assessment of predictor and criterion variables at the same time and place) can either inflate or deflate estimates of the relationship between two constructs. For example, transient mood states or consistency motif (i.e., the tendency to maintain consistency in responses to questions) may explain why some of the relationships between the constructs were significant only when measured at the same time point.

In summary, the study aimed to examine if this line of argument is supported by evidence using a longitudinal investigation. Specifically, we investigated the influence of human values on change in beliefs and concern about climate change (thus, statistically controlling beliefs and concern about climate change at Time 1). To investigate whether the longitudinal data in our study provide different results as their cross-sectional counterparts, we also reported the data analyzed separately for Time 1. The study was conducted in Italy. Italy was classified on the nineteenth location in the ranking according to the level of sustainable development of the European Union member states (Bluszcz 2016).

## Method

### Participants

Undergraduate/master students (*N* = 308; 233 female, 73 male) at an Italian public university participated for course credit. At the class sessions, after a brief description of the study objectives, students were invited to participate. Ten individuals who failed to complete both T1 and T2 assessment were removed from the sample, leaving a final sample of 298 individuals (228 female, 72 male). Students ranged in age from 21 to 62 years (*M* = 26.00, *SD* = 6.57).

### Measures

Participants were asked to fill out the same questionnaire at Time 1 and Time 2. In addition to the socio-demographic variables, the questionnaire included measures of perceived consequences of climate change, climate change risk perception, climate change skepticism, and human values. Table 1 shows the descriptive statistics and reliability of the scales. The mean of the items included in each measure was computed and each scale was scored such that higher scores indicate higher levels of the construct named.

**Table 1:** Correlations among and descriptive statistics for key study variables (N = 298)

	*M*	*SD*	α	1	2	3	4	5	6	7	8	9	10	11	12	13	14	15	16
1. Achievement (T1)	4.75	0.72	.68	–	.17	.34	.29	.19	.60	.06	−.02	.31	.31	.08	.09	.04	.03	−.04	−.05
2. Power (T1)	2.83	0.98	.77			−.18	.69	.10	.27	.02	−.18	.32	−.14	−.12	−.11	−.11	−.11	.19	.06
3. Universalism (T1)	4.70	0.72	.83				−.08	.29	.20	.26	.23	−.04	.62	.22	.31	.34	.35	−.22	−.25
4. Self-direction (T1)	3.78	0.95	.84					.22	.35	.06	−.13	.43	−.01	−.13	.00	−.05	−.02	.10	.02
5. Security (T1)	4.37	0.78	.72						.11	.55	.38	.14	.28	−.01	.01	.04	.09	−.03	.02
6. Stimulation (T1)	4.13	0.92	.75							.02	−.04	.51	.26	.01	−.01	.04	−.02	.01	.01
7. Conformity (T1)	4.41	0.76	.70								.45	.01	.35	−.01	−.02	.03	.06	.02	−.01
8. Tradition (T1)	3.57	0.75	.50									−.10	.29	−.03	.01	.08	.12	−.03	−.04
9. Hedonism (T1)	3.83	0.89	.75										.05	−.14	−.09	−.06	−.15	.19	.15
10. Benevolence (T1)	4.64	0.68	.73											.11	.13	.19	.20	−.16	−.16
11. Perceived consequences of climate change (T1)	8.00	1.26	.91												.61	.65	.57	−.46	−.40
12. Perceived consequences of climate change (T2)	8.09	1.21	.92													.61	.74	−.39	−.47
13. Climate change risk perception (T1)	2.26	1.29	.93														.71	−.51	−.48
14. Climate change risk perception (T2)	2.40	1.32	.94															−.48	−.58
15. Climate change skepticism (T1)	7.80	1.35	.79																.67
16. Climate change skepticism (T2)	7.87	1.36	.82																–

Correlations greater than ±.09 are significant at *p* < .05 (two-tailed test)

#### Perceived consequences of climate change

We used a set of nine items from a previous study (Bostrom et al. 2012) that includes perceived potential consequences of climate change to society, modified slightly to encompass perceived damages to the ecosystem (e.g., damages to the marine ecosystems). Item examples are “More and longer droughts in many parts of the world” and “More and larger storms in many parts of the world.” Respondents were asked to rate for each item how likely it is as a consequence of climate change. Responses were given on a 10-point scale, ranging from 1 = *extremely unlikely* to 10 = *extremely likely*.

#### Climate change risk perception

Climate change risk perception was assessed using four items from the study of Leiserowitz (2006). Specifically, this measure includes holistic concern (e.g., “How concerned are you about climate change?”) and perception of the seriousness and severity of future impacts of climate change (e.g., “How serious are the current impacts of climate change around the world?”). Response options ranged from 1 = *none* to 10 = *very*.

#### Climate change skepticism

Participants were asked to what extent they agree with four statements from the study of Whitmarsh (2011). Item examples are “Claims that human activities are changing the climate are exaggerated” and “Climate change is just a natural fluctuation in earth’s temperatures.” Scales range from 1 = *strongly disagree* to 10 = *strongly agree*. Higher scores indicated higher levels of climate change skepticism.

#### Human values

We used the Italian validated version of the Portrait Values Questionnaire (Capanna et al. 2005; Schwartz et al. 2001) to measure the 10 basic values. This questionnaire includes short verbal portraits of 40 different people. Each portrait defines a person’s objectives, aspirations, or desires that reflect a specific value. For each portrait, the respondents were asked to answer to the following question: “How much like you is this person?” using a response format ranging from 1 (*not like me at all*) to 6 (*very much like me*).

### Procedure

This research was conducted using a website accessible only to students. Each time participants logged into the study website, they read a consent form that explained the procedures of the study and their rights as participants (e.g., the voluntary and confidential nature of participation). After agreeing to take part and receiving their instructions, participants filled out the questionnaire at their convenience. Two months later, participants completed a second questionnaire with identical questions by again accessing the study website at their convenience. We chose a 2-month follow-up because the predominant causal influence between basic values and pro-environmental outcomes is supported in a short term perspective (Thøgersen and Ölander 2002).

### Statistical analysis

Missing values were less than 1% of the total number of cases, listwise deletion was preferred to imputation (Graham 2009). Because female participants were overrepresented in our sample, we also included gender as a predictor in our analysis. The analyses in our study were conducted using the structural equation modeling software IBM SPSS AMOS v.24. Specifically, we tested a Bayesian model examining the extent to which Time 1 values predict Time 2 beliefs and concern about climate change, while controlling for beliefs and concern about climate change at Time 1. Bayesian estimation offers several potential advantages over maximum likelihood estimation in structural equation modeling (Gelman et al. 2013). For instance, Bayesian methods provide more accurate results when parameters are not normally distributed or the sample size is small (e.g., Lee and Song 2004; Stegmueller 2013).

Unlike maximum likelihood estimation and hypothesis testing, using a Bayesian approach, each true model parameter is unknown and treated as a random variable that has a probability distribution. The distribution for the parameters is called a posterior distribution. Since to our knowledge, no study has investigated our set of hypotheses using a longitudinal design, we have performed Bayesian estimation for a model with the uniform prior distribution that Amos uses by default. By default, Amos assigns a uniform distribution from −3.4 × 10^−38^ to 3.4 × 10^38^ to each parameter. In a model with the uniform prior distribution for *θ*, *p*(*θ*) is completely flat. In addition, the posterior distribution is a re-normalized version of the likelihood of the observed data. Using the percentiles of the marginal posterior distribution, it is possible to compute the analogue of a confidence interval. We selected an interval that runs from the 2.5 percentile to the 97.5 percentile and forms a Bayesian 95% credible interval. Unlike conventional confidence intervals, the Bayesian credible intervals can be conceptualized as probability statements about the parameters themselves. When Prob (*a* ≤ *θ* ≤ *b*) = 0.95, we are 95% sure that the alternative hypothesis is true, that is, there is a probability of 95% that that the true value of *θ* lies between *a* and *b*. As a means of evaluating the quality of the fit of the model, we focused on posterior predictive *p* values (Lee and Song 2004). A posterior predictive *p* value near .5 indicate that the model is plausible, that is, little or any discrepancy between data generated by the model and the actual data itself (Gelman et al. 2013).

## Results

Table 1 displays correlations among human values and beliefs and concern about climate change. Power, self-direction, and tradition correlated with one or some of the measures of beliefs and concern about climate change. Hedonism was associated with almost all measures of beliefs and concern about climate change. Benevolence and universalism were associated with all measures of beliefs and concern about climate change.

Table 2 shows Bayesian path coefficients (and their 95% credibility intervals) predicting Time 2 beliefs and concern about climate change from Time 1 values controlling for the respective Time 1 beliefs and concern about climate change and gender. The 95% credibility intervals are often used in practice to decide whether the coefficients differ from zero. If the credibility interval does not include zero, the path coefficients can be seen as significant under conventional null hypothesis testing. Universalism at Time 1 was associated with Time 2 perceived consequences of climate change and climate change skepticism, but not climate change risk perception. Self-direction at Time 1 predicted Time 2 climate change risk perception and perceived consequences of climate change, but not Time 2 climate change skepticism. Hedonism at Time 1 only predicted Time 2 climate change risk perception. The other human values at Time 1 did not predict any of the measures of beliefs and concern about climate change at Time 2. This Bayesian model was correct based on the posterior predictive *p*-value test with a posterior predictive *p* value of .51, indicating that the model does adequately represent the observed data.

**Table 2:** Bayesian path coefficients (and 95% credible intervals) predicting Time 2 beliefs and concern about climate change from Time 1 values while controlling for gender and Time 1 beliefs and concern about climate change

	Perceived consequences of climate change (T2)	Climate change skepticism (T2)	Climate change risk perception (T2)
Achievement (T1)	−0.01 (−0.21, 0.19)	−0.04 (−0.26, 0.16)	0.00 (−0.21, 0.21)
Power (T1)	−0.16 (−0.31, 0.00)	−0.05 (−0.22, 0.11)	−0.08 (−0.24, 0.08)
Universalism (T1)	0.46* (0.27, 0.66)	−0.24* (−0.45, −0.01)	0.19 (−0.03, 0.41)
Self-direction (T1)	0.28* (0.12, 0.44)	−0.07 (−0.24, 0.10)	0.18* (0.01, 0.35)
Security (T1)	−0.10 (−0.27, 0.08)	0.16 (−0.02, 0.34)	0.05 (−0.13, 0.24)
Stimulation (T1)	−0.12 (−0.29, 0.04)	0.08 (−0.11, 0.24)	−0.05 (−0.21, 0.12)
Conformity (T1)	−0.06 (−0.24, 0.12)	−0.04 (−0.24, 0.15)	−0.02 (−0.21, 0.16)
Tradition (T1)	0.02 (−0.14, 0.19)	0.07 (−0.10, 0.25)	0.05 (−0.22, 0.11)
Hedonism (T1)	0.01 (−0.13, 0.15)	0.07 (−0.09, 0.23)	−0.19* (−0.34, −0.04)
Benevolence (T1)	−0.14 (−0.34, 0.06)	−0.01 (−0.24, 0.21)	0.03 (−0.19, 0.25)

The 95% credible intervals are in parentheses

* The 95% credible intervals did not include zero, indicating that the path coefficients can be seen as significant under conventional null hypothesis testing. Posterior predictive *p* value = .51

Table 3 displays Bayesian path coefficients predicting Time 1 beliefs and concern about climate change from Time 1 values controlling for gender. Universalism at Time 1 was related to Time 2 perceived consequences of climate change, climate change skepticism, and climate change risk perception. Hedonism at Time 1 predicted Time 1 perceived consequences of climate change and climate change skepticism but did not predict climate change risk perception. Power at Time 1 only predicted Time 1 climate change skepticism. The other human values at Time 1 did not predict any of the measures of beliefs and concern about climate change at Time 2. A posterior predictive *p* value of .49 indicate that the Bayesian model was plausible.

**Table 3:** Bayesian path coefficients (and 95% credible intervals) predicting Time 1 beliefs and concern about climate change from Time 1 values while controlling for gender

	Perceived consequences of climate change (T1)	Climate change skepticism (T1)	Climate change risk perception (T1)
Achievement (T1)	0.07 (−0.19, 0.32)	0.00 (−0.26, 0.27)	−0.21 (−0.48, 0.06)
Power (T1)	0.00 (−0.19, 0.19)	0.24* (0.02, 0.46)	−0.06 (−0.28, 0.15)
Universalism (T1)	0.38* (0.12, 0.67)	−0.30* (−0.57, −0.01)	0.76* (0.50, 1.02)
Self-direction (T1)	−0.12 (−0.32, 0.09)	−0.12 (−0.34, 0.10)	0.09 (−0.14, 0.32)
Security (T1)	0.03 (−0.21, 0.27)	−0.08 (−0.32, 0.18)	−0.05 (−0.29, 0.19)
Stimulation (T1)	0.07 (−0.14, 0.28)	−0.07 (−0.30, 0.16)	0.08 (−0.16, 0.31)
Conformity (T1)	−0.05 (−030, 0.19)	0.16 (−0.09, 0.40)	−0.10 (−034, 0.14)
Tradition (T1)	−0.16 (−0.38, 0.07)	0.07 (−0.15, 0.30)	0.05 (−0.19, 0.27)
Hedonism (T1)	−0.21* (−0.40, −0.03)	0.30* (0.09, 0.51)	−0.07 (−0.26, 0.12)
Benevolence (T1)	−0.02 (−0.31, 0.26)	−0.12 (−0.39, 0.17)	−0.06 (−0.33, 0.23)

The 95% credible intervals are in parentheses

* The 95% credible intervals did not include zero, indicating that the path coefficients can be seen as significant under conventional null hypothesis testing. Posterior predictive *p* value = .49

## Discussion

The current study aimed to investigate the extent to which human values measured at Time 1 and beliefs and concern about climate change measured at Time 2 are related when controlling for Time 1 beliefs and concern about climate change (auto-regressive effects). Our findings did not lend strong support to the first and the second hypotheses. Specifically, we found that (1) among self-transcendent values, universalism (but not benevolence) was related to higher levels of perceived consequences of climate change (0.46, *CI* 0.27, 0.66) and lower levels of climate change skepticism (−0.24, *CI* −0.45, −0.01); (2) among self-enhancing values, achievement did not relate to lower levels of perceived consequences of climate change (−0.01, *CI* −0.21, 0.19) and climate change risk perception (0.00, *CI* −0.21, 0.21) and higher levels of climate change skepticism (−0.04, *CI* −0.26, 0.16). Also, power was not associated with lower levels of perceived consequences of climate change (−0.16, *CI* −0.31, 0.00) and climate change risk perception (−0.08, *CI* −0.24, 0.08) and higher levels of climate change skepticism (−0.05, *CI* −0.22, 0.11). The relationship between hedonism at Time 1 and climate change risk perception at Time 2 was significant and, as expected, had a negative sign (−0.19, *CI* −0.34, −0.04). However, hedonism did not predict perceived consequences of climate (0.01, *CI* −0.13, 0.15) and climate change skepticism (0.07, *CI* −0.09, 0.23).

Previous research indicated that the dimension contrasting self-transcendent versus self-enhancement values is more related to different kinds of environmental beliefs than the openness to change versus conservatism (e.g., de Groot and Steg 2008, 2010; Corner et al. 2014; Stern and Dietz 1994; Nordlund and Garvill 2002). The results of the present study are in line with studies indicating that the self-enhancement value has no influence on beliefs and concern about climate change or pro-environmental behavior (e.g., Slimak and Dietz 2006; Thøgersen and Ölander 2002). In other words, participants who held stronger self-transcendent values were more likely to report higher levels of concern about climate change than people who held weaker self-transcendent values, irrespective of their self-enhancement value. This pattern of findings can be explained by the fact that people can express multiple and divergent values. In their investigation of the relationship between values and their consumer behavior, Gatersleben et al. (2009) found that environmental concern and materialistic values can coexist.

In our opinion, the conflict between self-enhancement values and beliefs and concern about climate change cannot entirely explain the negative association between hedonism and climate change risk perception. Hedonism reflects pleasure or gratification for oneself and shares elements of both self-enhancement and openness to change. People who strongly desire immediate pleasure and sensory gratification for oneself may be less likely to appraise the environment as threatening (Kaptan et al. 2013).

The results of the present study do not provide evidence that self-transcendence values are consistently associated with beliefs and concern about climate change because benevolence did not relate to any of the measures of beliefs and concern about climate change. Previous studies revealed that universalism values are more strongly related to environmentally responsible beliefs and behavior than benevolence values (e.g., Axelrod 1994; Gärling 1999; Schultz and Zelezny 1999). The results of the present study indicated that when it comes to beliefs and concern about climate change, benevolence values are not relevant. This is interesting given that benevolence and universalism values were strongly interrelated (see Table 1). One reason for this result could be that concern for the welfare of people with whom one is in frequent personal contact (i.e., benevolence) and the welfare of all people and nature (i.e., universalism) may sometimes conflict with each other when it comes to pro-environmental outcomes (Thøgersen and Ölander 2002). For example, energy consumption is considered one of the major issues under the changing climate (Intergovernmental Panel on Climate Change 2014): the concern for the welfare of close others in everyday interaction may require energy consumption (e.g., electric energy consumption, oil consumption) that is in conflict with the concern for nature and environment.

As concerns our research question, security, conformity, tradition, and stimulation values did not play a significant role in explaining engagement with climate change. However, self-direction predicted later perceived consequences of climate change and climate change risk perception. Self-direction stems from organismic needs for control and mastery and involves a preference for autonomy, creativity, independent thought and action, and freedom. Self-direction may denote people who feel particularly empowered or with high levels of internal locus of control. A sense of empowerment and personal responsibility (as opposed to disinterest associated with external locus of control and fatalism) has been found to be related to engagement to the risks posed by climate change (Langford 2002). In addition, extant literature indicates that people with high levels of self-direction are more likely to control distressing emotions about a terror risk (Kaptan et al. 2013).

It is interesting to note that the pattern of findings concerning self-direction did not emerge in the cross-sectional analysis. The inconsistency between the present study and the previous research can be ascribed in part to the different methodology—that is, cross-sectional versus longitudinal. The correlation analyses (Table 1) as well as the use of a correlational design (Table 3) revealed different findings. Different factors are likely to account for the difference between cross-sectional versus longitudinal studies. For instance, common method variance is more likely to either inflates or deflates regression estimates in cross-sectional studies than in longitudinal studies (Podsakoff et al. 2012). Moreover, in the present study, we controlled for the auto-regressive effects of the outcome variables (i.e., beliefs and concern about climate change) on themselves, measured on subsequent occasions. By partially out the auto-regressive effect, the relationships found in the present study reflect the influence of the scores of human values on the change in beliefs and concern about climate change. Therefore, it is possible to draw firmer inferences or conclusions about causal linkages.

Our findings have implications for effective communication strategies about climate change aimed at promoting public engagement. According to Corner et al. (2014), in the last years, two approaches in values-based climate change campaigning have been proposed: campaigns oriented towards self-transcendent values and campaigns based on value-neutrality provided by social marketing. Following the social marketing approach, efforts are made to match campaign messages to the values held by the target populations (even if they are incongruent with environmental engagement). The social marketing approach is not entirely convincing for various reasons, the most prominent of which is that it does not engage the public on climate change (Corner et al. 2014). The findings of the present study suggest that, to build support for ambitious policy changes and interventions necessary to address climate change, universal and self-direction values (not all the self-transcendent values including benevolence) should be considered when designing the message. This does not mean that a focus on self-enhancing values is not effective to promote specific pro-environmental behaviors. However, for ‘bigger than self’ issues as climate change, the activation of self-enhancing values is likely to be ineffective in the long term (Corner et al. 2014). Finally, communication strategies about climate change may benefit from stimulation of one’s own thought and judgment as this is compatible with attaining the goals of self-direction.

Several potential limitations to this research need to be addressed. First, the use of self-reported measures raises concerns about the possibility of response biases. However, it could be argued that when it comes to address climate change, people’s perceptions and evaluations are crucial because they can drive or accept important policy changes and interventions (Corner et al. 2014). Second, we collected data from a convenience sample—undergraduate/master students. Although various subgroups of the general public may hold different levels of beliefs and concern about climate change, there is no evidence that the relationships between human values and beliefs and concern about climate change differ between segments of the population. It should be noted that the involvement of students allowed us to attain high follow-up response rates. In longitudinal studies, this is essential to reduce potential non-response bias. Third, we note that the effect sizes found in the present research are considered to be small by conventional standards (Cohen 1988). Such small effect sizes are common in longitudinal studies when accounting auto-regressive effects of the outcome variables. Moreover, we note that, based on Values-Beliefs-Norms Theory (Stern and Dietz 1994), such small effects could be expected because human values are thought to indirectly influence public engagement with climate change (Dietz et al. 2007). Fourth, for our Bayesian analysis we used the default uniform prior distribution of Amos. Previous knowledge about the value of these parameters is missing because, as far as we know, no longitudinal investigation has examined the influence of Time 1 values on Time 2 beliefs and concern about climate change while controlling for gender and Time 1 beliefs and concern about climate change. The use of the default uniform prior distribution introduces as little information as possible and spreads its probability over a very wide range of parameter values. A prior distribution quantifies the researcher’s information about the parameters before observing the data. However, a Bayesian analysis tend to change very little when using such prior information, unless the sample is unusually small or if a model or a prior distribution is strongly contradicted by the data.

In summary, we found support for the influence of universalism as well as self-direction values on beliefs and concern about climate change. The other human values postulated in Value Theory (Schwartz 1992, 1994) did not seem to play an important role with the exception of a marginal effect of hedonism. We suggest that people who identify strongly with self-enhancing values are not necessarily less engaged with climate change probably because they possess a range of different and sometimes conflicting values. A focus on universalism and self-direction values seems to be a more fruitful approach to inspire public engagement with climate change. Finally, future research should focus on every human value rather than on global dimensions such as conservation versus openness to change or self-transcendence versus self-enhancement.
